# Synthesis of 3-(quinolin-2-yl)- and 3,6-bis(quinolin-2-yl)-9*H*-carbazoles

**DOI:** 10.3762/bjoc.6.108

**Published:** 2010-10-08

**Authors:** Yang Li, Wentao Gao

**Affiliations:** 1 Institute of Superfine Chemicals, Bohai University, Jinzhou 121000, China

**Keywords:** acetylcarbazole, β-aminoaldehyde, β-aminoketone, Friedländer condensation, sodium ethoxide

## Abstract

A simple and efficient synthesis of novel 3-(quinolin-2-yl)- and 3,6-bis(quinolin-2-yl)-9*H*-carbazoles, utilizing sodium ethoxide as a catalyst via a Friedländer condensation reaction between 3-acetyl-9-ethyl-9*H*-carbazole or 3,6-diacetyl-9-ethyl-9*H*-carbazole and β-aminoaldehydes or β-aminoketones is described. All of the title compounds were obtained in good yields of 52–72% and their structures were confirmed by IR, ^1^H NMR, MS, and elemental analysis.

## Introduction

Nitrogen-containing heterocycles are a very important group of organic compounds because of their wide application in medicine, agriculture, and technology. Among these, quinoline and carbazole derivatives are of significant synthetic interest due to their diverse range of biological activities. Compounds containing a quinoline framework have been found applications as pharmaceuticals and agrochemicals, as well as being general synthetic building blocks [1–3]. Industrial, biological, and synthetic significance places this scaffold in a prestigious position. Studies on new quinoline derivatives appear frequently in the chemical literature. Therefore, significant effort continues to be directed toward the development of new quinolines . In particular, there is much current interest in the quinoline ring system especially in the area of medicinal chemistry, and moreover it is a ubiquitous sub-structure found in many biologically active natural products [4–8]. Carbazole-based compounds are also embodied in many naturally occurring products and these too display a wide variety of biological effects such as anti-tumor [9–10], anti-oxidative [11], anti-inflammatory, and anti-mutagenic activities [12–13]. In addition, their derivatives are widely used as building blocks for new organic materials and play a very important role in electroactive and photoactive materials. They are also considered to be potential candidates for electronic devices, such as color displays, organic semiconductor lasers, and solar cells because of their reversible electrochemical oxidation [14–20]. Currently, there is a strong interest in the synthesis of novel heteroarylcarbazole derivatives due to their intriguing structural features and promising biological activities [21–24]. Most heteroarylcarbazoles reported in the literature contain a common heterocyclic ring moiety fused with a carbazole such as pyridocarbazoles [25], thienocarbazole [26], quino and chromenocarbazoles [27], pyranocarbazoles, pyrrolocarbazoles [28], indolocarbazoles [29], and synthetic analogues thereof. However, to the best of our knowledge, there are very few reports where the heteroaryl moiety is substituted with a carbazole unit and hence the synthesis of such compounds is desirable. In this regard, Meesala et al. [30] recently described a short and facile route to the synthesis of new 3,6-bis(pyrazol-4-yl)carbazoles from 3,6-diacetylcarbazoles through a Vilsmeier reaction. Later, Chaitanya et al. [31] reported a new synthesis of 3-(3-nitrochromenyl)carbazoles, 3,6-bis(3-nitrochromenyl)carbazoles under solvent-free conditions by the reaction of β-nitrovinylcarbazole or bis(β-nitrovinyl)carbazole with salicylaldehydes.

In light of these findings, and in view of the prominent role structural diversity plays in medicinal and combinatorial chemistry, we felt that there was a real need for the synthesis of some new prototypes combining both the carbazole ring system and quinoline moiety in the same molecule. These could be vitally important for pharmacological studies or in creating new medicinal properties. Therefore, in continuation of our studies on the synthesis of novel and interesting quinolyl-substituted heterocycles [32–34], we report herein the synthesis of novel 3-(quinolin-2-yl)- and 3,6-bis(quinolin-2-yl)-9*H*-carbazoles.

## Results and Discussion

**Scheme 1:**
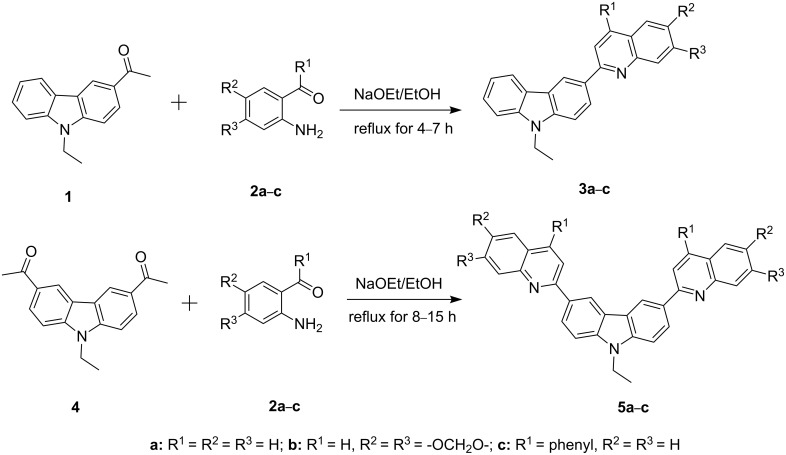
Synthetic route of the title compounds **3a–5c**.

In order to construct the desired quinolyl-substituted carbazoles, we devised a route that made use of a Friedländer condensation reaction between the readily available 3-acetyl-9-ethyl-9*H*-carbazole (**1**) or 3,6-diacetyl-9-ethyl-9*H*-carbazole (**4**) and β-aminoaldehydes or β-aminoketone (**2a–c**) as shown in Scheme 1.

The starting materials **1** and **4** of our study were readily prepared according to the methods described in literature [35]. For the construction of the quinoline ring, the Friedländer quinoline synthesis appears to be the most simple and straightforward approach available to chemists, compared to other possible methods such as the Skraup, Doebner–von Miller, and Combes reactions [36–42]. Conventionally, the Friedländer quinoline synthesis can be achieved by the use of a variety of Brønsted acid catalysts, such as hydrochloric acid, perchloric acid, sulfuric acid, *p*-toluene sulfonic acid, sulfamic acid, phosphoric acid, and trifluoroacetic acid [43–45]. However, many of these procedures are not completely satisfactory with regard to operational simplicity, cost of the reagent, drastic reaction conditions, and relatively low yields. Some recent protocols reported for the synthesis of quinolines involve the use of catalysts such as SnCl_2_ [46], I_2_ [47], montmorillonite-KSF [48], ionic liquids [49], Bi(OTf)_3_ [50], Y(OTf)_3_ [51], silver dodecatungstophosphate (Ag_3_POW_12_O_40_) [52], silica sulfuric acid [53], sulfamic acid [54], neodymium nitrate [55], etc. However, these methods suffer from the serious drawbacks of harsh reaction conditions, use of expensive catalysts, long reaction times, etc. Ionic liquids have emerged as effective solvents for green chemical processes. However, the high cost of most conventional ionic liquids and their toxicity has limited their use. Recently, Yang et al. [56] reported a Friedländer condensation reaction for the facile synthesis of various poly-substituted quinolines under mild conditions catalysed by sodium ethoxide. In connection with our studies, we envisioned that the procedure could also be applied to the reaction of 3-acetyl-9-ethyl-9*H*-carbazole or 3,6-diacetyl-9-ethyl-9*H*-carbazole with 2-aminoaldehydes or 2-aminoketones, from which the quinolycarbazoles 9-ethyl-3-(quinolin-2-yl)-9*H*-carbazoles and 9-ethyl-3,6-bis(quinolin-2-yl)-9*H*-carbazoles might be synthesized. Indeed, this was found to be the case as shown in Scheme 1. The Friedländer condensation reaction of 3-acetyl-9-ethyl-9*H*-carbazole (**1**) with 1 molar equivalent of β-aminoaldehydes or β-aminoketone (**2a–c**) in the presence of 1 molar equivalent of sodium ethoxide in anhydrous ethanol under reflux for 6–8 h afforded the corresponding products (**3a–c**) in good yields of 59–72%. The ease of isolation of all the products was notable; after aqueous work-up, the products were isolated as the main products. In addition, it is noteworthy that the use of 1 molar equivalent of the catalyst EtONa was sufficient to promote the reaction and that there were no improvements in reaction rates and yields by increasing the amount of EtONa or by the use of other bases such as NaOH or K_2_CO_3_. Similarly, 3,6-diacetyl-9-ethyl-9*H*-carbazole (**4**) reacted with 2 molar equivalents of **2a–c** in the presence of sodium ethoxide to yield the desired products **5a–c** in good yields (66%, 58%, and 52%, respectively). The yields and melting points of all the synthesized compounds **3a–5c** are listed in Table 1.

**Table 1:** Syntheses of 3-(quinolin-2-yl)- and 3,6-bis(quinolin-2-yl)-9*H*-carbazoles (**3a–5c**).

Entry	Substrate	Time (h)	Product		Yield (%)^a^	Mp (°C)

1	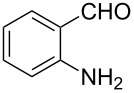	4	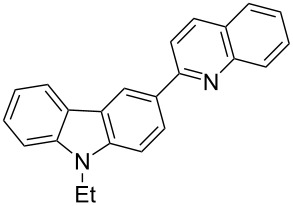	**3a**	72	116–117 Lit [57]: 116–117
2	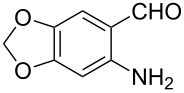	5	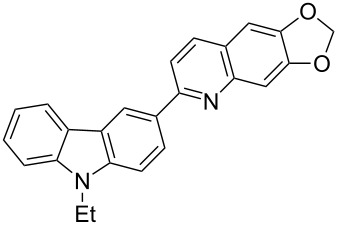	**3b**	65	170–172
3	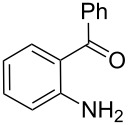	7	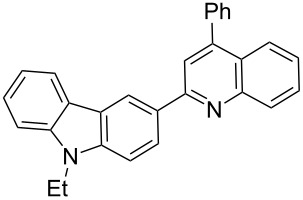	**3c**	59	210–211
4	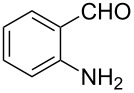	8	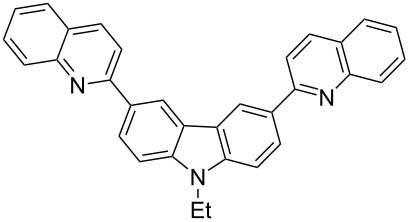	**5a**	66	208–209
5	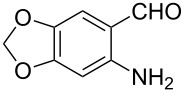	11	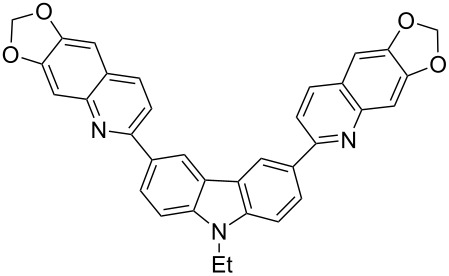	**5b**	58	245–247
6	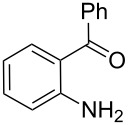	15	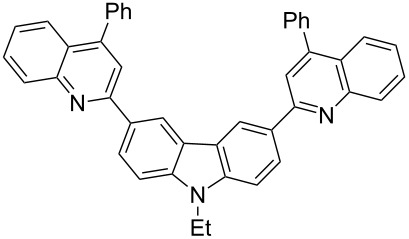	**5c**	52	260–262

^a^Isolated yield.

As shown in Table 1, both 9-ethyl-9*H*-carbazoles bearing acetyl and diacetyl substituents afforded the corresponding products in good yields. Additionally, some reaction trends were also noted. Thus, the use of 3,6-diacetyl-9-ethyl-9*H*-carbazole led to slightly lower yields of products when compared to 3-acetyl-9-ethyl-9*H*-carbazole and required relatively longer reaction times (Entries 4–6). On the other hand, in going from **2a** to **2c** with either of the starting carbazoles, the yields of both sets of products, i.e., **3a–c** (Entries 1–3) and **5a–c** (Entries 4–6) gradually decreased, whilst the corresponding reaction times gradually increased. This may be attributed to the reduced electrophilicity of the carbonyl groups in **2b** and **2c** as a result of the electron-rich nature of the methylenedioxy group or the phenyl ring. This observation is consistent with the proposed mechanism which involves the enolate anion formed in the basic medium attacking by the carbonyl carbon of the *ortho*-aminoarylaldehyde [56].

It is worth noting here that only compound **3a** is known and its melting point and proton NMR spectral data are identical with the literature data [57], further corroborating the assigned structure. Compounds **3b–5c** are novel and their structures were established with the help of spectroscopic data and elemental analysis. For example, the IR spectrum of **3b** revealed no absorption for amino or carbonyl groups. Its ^1^H NMR spectrum showed no signals attributable to an amino group but contained signals for eleven aromatic protons between δ 7.07–8.86 ppm and for two methylenedioxy protons at δ 6.11 ppm as well as signals from the ethyl group, which is consistent with the attachment of the nascent quinoline ring moiety to the carbazole substrate. Finally, the structure was confirmed by its mass spectrum through the appearance of a quasi-molecular ion peak at *m/z* 367.1 ([M+H]^+^). The obtained elemental analysis values are in agreement with theoretical values. The other synthesized compounds exhibited similar spectral characteristics.

## Conclusion

A straightforward synthesis of 3-(quinolin-2-yl)- and 3,6-bis(quinolin-2-yl)-9*H*-carbazoles (**3a–5c**) in good yields is reported. These molecules should allow us, in the future, to investigate structure-activity relationships in various biotests.

## Experimental

Melting points (uncorrected) were determined by using a WRS-1B melting point apparatus. The ^1^H NMR (400 MHz) spectra were recorded on a Bruker AVANCE 400 NMR spectrometer at 400 MHz with TMS as the internal standard. The mass spectra were determined using a MSD VL ESI1 spectrometer. Elemental analyses were performed with an Elementar Vario EL-III element analyzer. The progress of reactions was monitored by thin-layer chromatography (TLC) on silica gel GF254 using ethyl acetate/petroleum ether (1:4) as eluent.

**General procedure for the synthesis of 9-ethyl-3-(quinolin-2-yl)-9*H*-carbazole (3a–c).** To a solution of 3-acetyl-9-ethyl-9*H*-carbazole (**1**) (0.24 g, 1 mmol) and the required β-amino carbonyl compound, 2-aminobenzaldehyde, 2-aminobenzophenone or 2-amino-4,5-methylenedioxybenzaldehyde (**2a–c**), (1 mmol) in 5 mL of absolute EtOH, was added sodium ethoxide (0.07 g, 1 mmol). The resulting mixture was heated under reflux for 4–7 h. After the reaction was complete (TLC), the mixture was cooled to room temperature, poured into water and filtered to give the crude product, which was then purified by silica gel column chromatography with ethyl acetate/petroleum ether (1:6) as eluent. The reaction times, yields and melting points are listed in Table 1.

**9-Ethyl-3-(quinolin-2-yl)-9*H*-carbazole (3a).** This compound was obtained as a yellow solid, IR (KBr) ν/cm^−1^: 3049, 2978, 1596, 1551, 1493, 1473, 1452, 1437, 1379, 1237, 1141, 879, 808, 745; ^1^H NMR (CDCl_3_) δ (ppm): 8.93 (d, *J* = 1.5 Hz, 1H, ArH), 8.35 (dd, *J* = 8.5, 2.0 Hz, 1H, ArH), 8.20–8.24 (m, 3H, ArH), 8.03 (d, *J* = 8.5 Hz, 1H, ArH), 7.82 (dd, *J* = 8.5, 1.0 Hz, 1H, ArH), 7.53 (d, *J* = 8.5 Hz, 1H, ArH), 7.48–7.52 (m, 2H, ArH), 7.44 (d, *J* = 8.0 Hz, 1H, ArH), 7.35 (dd, *J* = 7.0, 1.5 Hz, 1H, ArH), 7.27 (dd, *J* = 8.0, 7.5 Hz, 1H, ArH), 4.42 (q, *J* = 7.5 Hz, 2H, CH_2_), 1.47 (t, *J* = 7.5 Hz, 3H, CH_3_); MS (ESI, *m/z*): 323.1 [M+H]^+^; Anal. Calcd for C_23_H_18_N_2_: C, 85.68; H, 5.63; N, 8.69. Found: C, 85.21; H, 5.68; N, 8.45.

**9-Ethyl-3-(6,7-methylenedioxyquinolin-2-yl)-9*H*-carbazole (3b).** This compound was obtained as a yellow solid, IR (KBr) ν/cm^−1^: 3062, 2973, 2897, 1594, 1541, 1494, 1458, 1393, 1339, 1252, 1235, 1173, 1142, 1125, 1039, 927, 853, 746; ^1^H NMR (CDCl_3_) δ (ppm): 8.86 (d, *J* = 1.32 Hz, 1H, ArH), 8.27 (dd, *J* = 8.5, 1.6 Hz, 1H, ArH), 8.22 (d, *J* = 7.7 Hz, 1H, ArH), 8.02 (d, *J* = 8.5 Hz, 1H, ArH), 7.86 (d, *J* = 8.5 Hz, 1H, ArH), 7.45–7.52 (m, 4H, ArH), 7.26 (s, 1H, ArH), 7.07 (s, 1H, ArH), 6.11 (s, 2H, OCH_2_O), 4.43 (q, *J* = 7.2 Hz, 2H, CH_2_), 1.47 (t, *J* = 7.2 Hz, 3H, CH_3_); MS (ESI, *m/z*): 367.1 [M+H]^+^; Anal. Calcd for C_24_H_18_N_2_O_2_: C, 78.67; H, 4.95; N, 7.65. Found: C, 78.35; H, 4.80; N, 7.53.

**9-Ethyl-3-(4-phenylquinolin-2-yl)-9*H*-carbazole (3c).** This compound was obtained as a yellow solid, IR (KBr) ν/cm^−1^: 3057, 2925, 2854, 1589, 1542, 1495, 1473, 1457, 1442, 1385, 1232, 1160, 1133, 879, 771; ^1^H NMR (CDCl_3_) δ (ppm): 8.97 (s, 1H, ArH), 8.41 (d, *J* = 8.4 Hz, 1H, ArH), 8.29 (d, *J* = 8.7 Hz, 1H, ArH), 8.23 (d, *J* = 7.8 Hz, 1H, ArH), 7.92 (d, *J* = 8.7 Hz, 1H, ArH), 7.75 (dd, *J* = 8.1, 7.2 Hz, 1H, ArH), 7.64 (d, *J* = 8.1 Hz, 1H, ArH), 7.44–7.59 (m, 7H, ArH), 7.27–7.31 (m, 3H, ArH), 4.45 (q, *J* = 7.2 Hz, 2H, CH_2_), 1.49 (t, *J* = 7.2 Hz, 3H, CH_3_); MS (ESI, *m/z*): 399.1 [M+H]^+^; Anal. Calcd for C_29_H_22_N_2_: C, 87.41; H, 5.56; N, 7.03. Found: C, 87.13; H, 5.61, N, 6.79.

**General procedure for the synthesis of 3,6-bis(quinolin-2-yl)-9-ethyl-9*H*-carbazole (5a–c).** To a solution of 3,6-diacetyl-9-ethyl-9*H*-carbazole (**4**) (0.28 g, 1 mmol) and the required β-amino carbonyl compound, 2-aminobenzaldehyde, 2-aminobenzophenone or 2-amino-4,5-methylenedioxybenzaldehyde (**2a–c**), (2 mmol) in 5 mL of absolute EtOH was added sodium ethoxide (0.14 g, 2 mmol). The resulting mixture was heated under reflux for 8–15 h. After the reaction was complete (TLC), the mixture was cooled to room temperature, poured into water and filtered to give the crude product, which was then purified by silica gel column chromatography with ethyl acetate/petroleum ether (1:6) as eluent. The reaction times, yields and melting points are listed in Table 1.

**3,6-Bis(quinolin-2-yl)-9-ethyl-9*H*-carbazole (5a).** This compound was obtained as a white solid, IR (KBr) ν/cm^−1^: 3050, 2974, 2929, 1592, 1552, 1488, 1434, 1381, 1329, 1286, 1242, 1178, 1148, 1129, 1043, 973, 890, 786, 763; ^1^H NMR (CDCl_3_) δ (ppm): 9.05 (d, *J* = 1.5 Hz, 2H, ArH), 8.39 (dd, *J* = 8.4, 1.5 Hz, 2H, ArH), 8.17–8.25 (m, 4H, ArH), 8.06 (d, *J* = 8.7 Hz, 2H, ArH), 7.80 (dd, *J* = 8.1, 1.8 Hz, 2H, ArH), 7.72 (dd, *J* = 8.4, 1.5 Hz, 2H, ArH), 7.48–7.56 (m, 4H, ArH), 4.45 (q, *J* = 7.2 Hz, 2H, CH_2_), 1.49 (t, *J* = 7.2 Hz, 3H, CH_3_); MS (ESI, *m/z*): 450.4 [M+H]^+^; Anal. Calcd for C_32_H_23_N_3_: C, 85.50; H, 5.16; N, 9.35. Found: C, 85.09; H, 5.21; N, 9.46.

**3,6-Bis(6,7-methylenedioxyquinolin-2-yl)-9-ethyl-9*H*-carbazole (5b).** This compound was obtained as a yellow solid, IR (KBr) ν/cm^−1^: 3052, 2958, 2925, 2853, 1595, 1521, 1492, 1470, 1403, 1352, 1329, 1264, 1231, 1174, 1126, 1074, 1041, 961, 934, 861, 811,777; ^1^H NMR (CDCl_3_) δ (ppm): 8.90 (d, *J* = 1.5 Hz, 2H, ArH), 8.25 (dd, *J* = 7.0, 1.5 Hz, 2H, ArH), 7.98 (d, *J* = 7.0 Hz, 2H, ArH), 7.49 (d, *J* = 7.5 Hz, 2H, ArH), 7.38 (d, *J* = 7.5 Hz, 2H, ArH), 7.19 (s, 2H, ArH), 7.02 (s, 2H, ArH), 6.06 (s, 4H, OCH_2_O), 4.39 (q, *J* = 7.0 Hz, 2H, CH_2_), 1.41 (t, *J* = 7.0 Hz, 3H, CH_3_); MS (ESI, *m/z*): 538.1 [M+H]^+^; Anal. Calcd for C_34_H_23_N_3_O_4_: C, 75.97; H, 4.31; N, 7.82. Found: C, 76.19; H, 4.17; N, 7.72.

**3,6-Bis(4-phenylquinolin-2-yl)-9-ethyl-9*H*-carbazole (5c).** This compound was obtained as a yellow solid, IR (KBr) ν/cm^−1^: 3049, 2976, 2932, 1587, 1544, 1488, 1446, 1409, 1367, 1346, 1275, 1231, 1174, 1127, 1074, 1022, 919, 878, 773; ^1^H NMR (CDCl_3_) δ (ppm): 9.05 (d, *J* = 1.2 Hz, 2H, ArH), 8.47 (dd, *J* = 8.4, 1.5 Hz, 2H, ArH), 8.29 (d, *J* = 8.4 Hz, 2H, ArH), 8.02 (s, 2H, ArH), 7.91 (d, *J* = 8.4 Hz, 2H, ArH), 7.71–7.77 (m, 6H, ArH), 7.43–7.65 (m, 10H, ArH), 4.48 (q, *J* = 7.2 Hz, 2H, CH_2_), 1.52 (t, *J* = 7.2 Hz, 3H, CH_3_); MS (ESI, *m/z*): 602.1 [M+H]^+^; Anal. Calc for C_44_H_31_N_3_: C, 87.82; H, 5.19; N, 6.98. Found: C, 88.27; H, 5.07; N, 6.90.

## Supporting Information

Supporting Information features ^1^H NMR spectra of the synthesized 3-(quinolin-2-yl)- and 3,6-bis(quinolin-2-yl)-9*H*-carbazoles.

File 1 ^1^H NMR spectra of the title compounds **3a–5c**.
