# Supplementary material for: Synthesis of 3-(quinolin-2-yl)- and 3,6-bis(quinolin-2-yl)-9H-carbazoles
Source: Beilstein J Org Chem. 2010 Oct 8;6:966–72. doi: 10.3762/bjoc.6.108 (PMC2981831; doi:10.3762/bjoc.6.108)
Supplement: File 1 — 1H NMR spectra of the title compounds 3a–5c. [file Beilstein_J_Org_Chem-06-966-s001.pdf]

Supporting Information

for

Synthesis of 3-(quinolin-2-yl)- and 3,6-bis(quinolin-2-yl)-9*H*-carbazoles

Yang Li and Wentao Gao*

Address: Institute of Superfine Chemicals, Bohai University, Jinzhou 121000, China

Email: Wentao Gao - bhuzh@163.com

* Corresponding author

^{1}H NMR spectra of the title compounds **3a–5c**

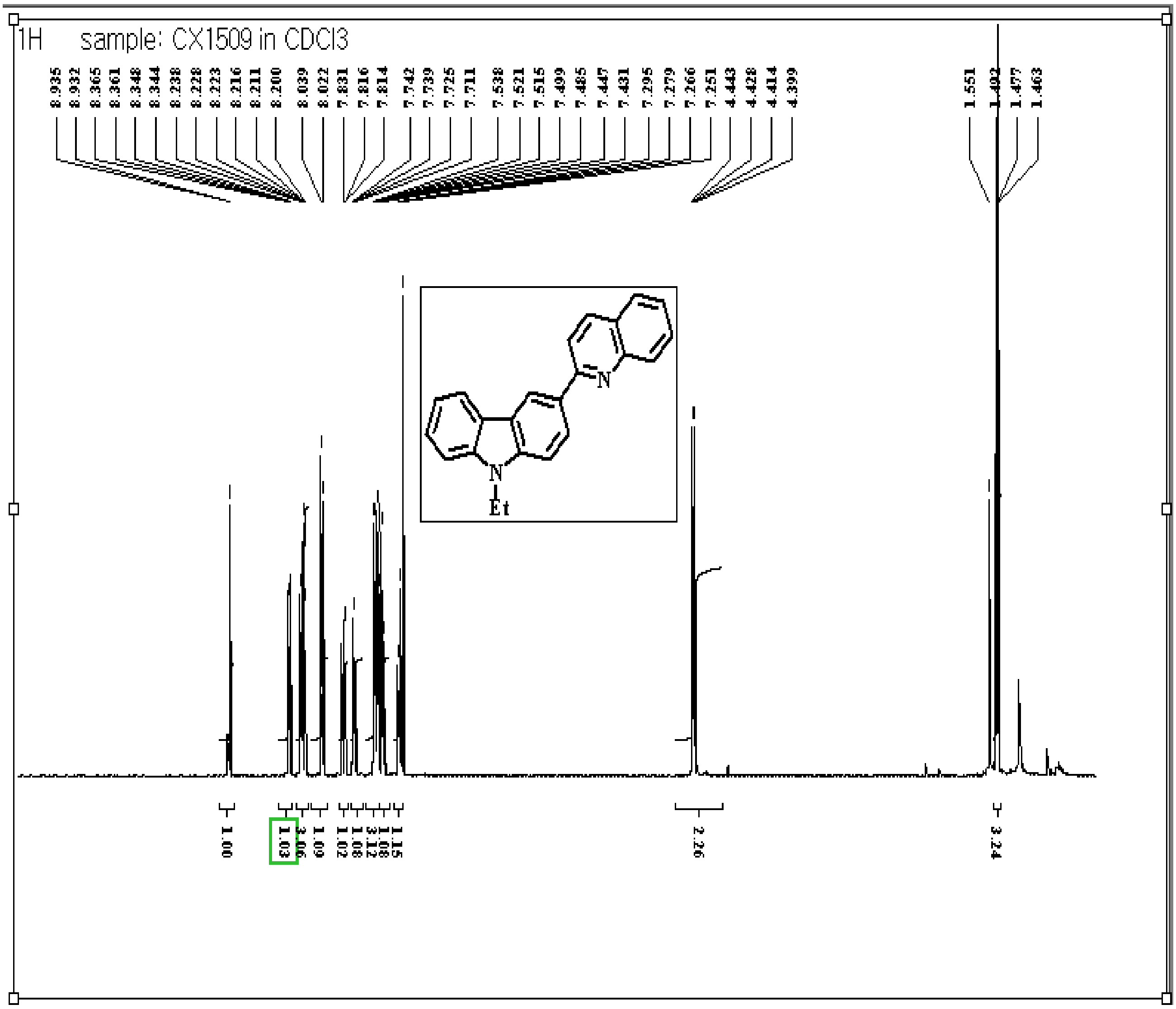

Figure S1: ^{1}H NMR spectrum of **3a.**

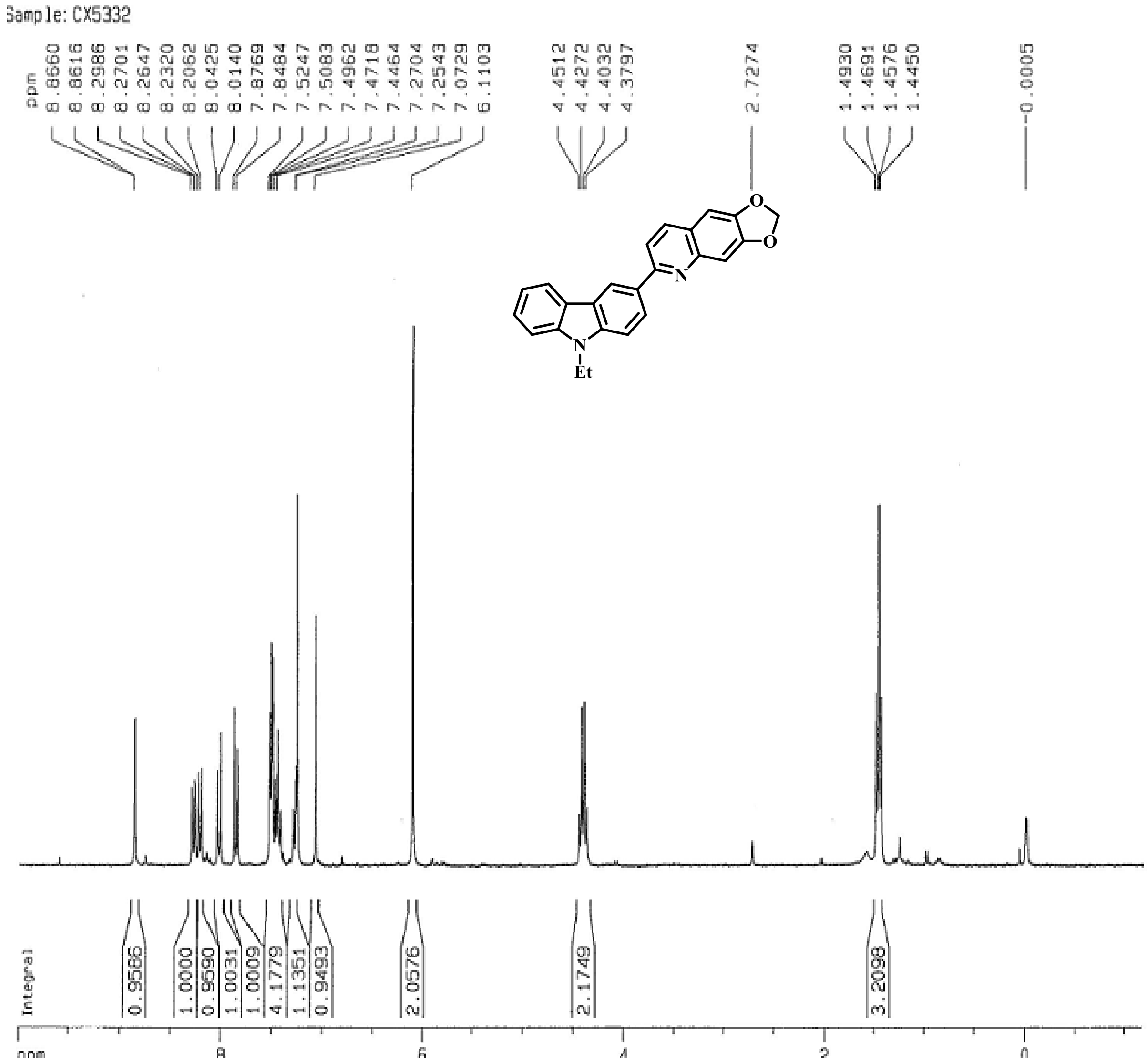

Figure S2: ^{1}H NMR spectrum of **3b.**

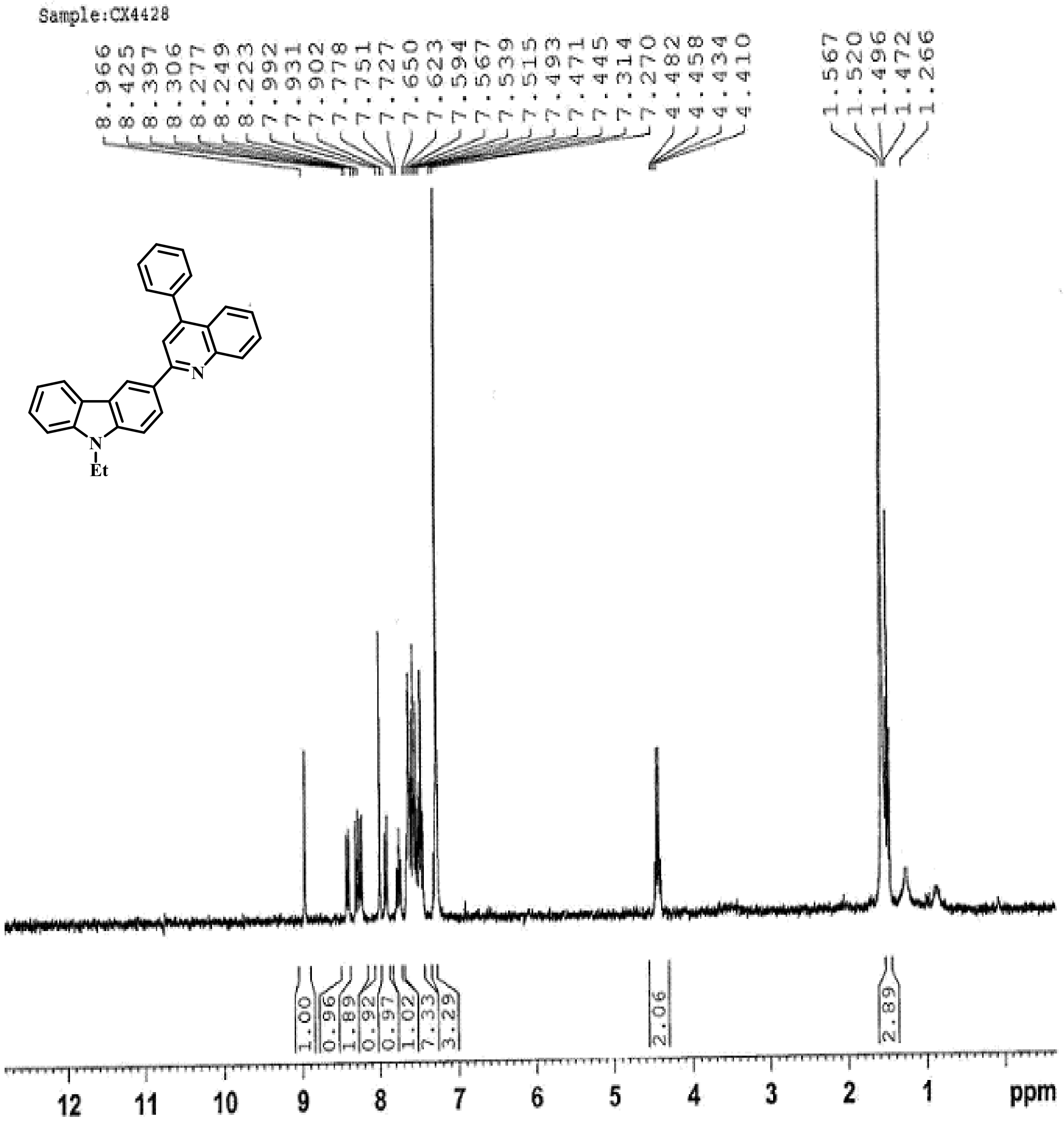

Figure S3: ^{1}H NMR spectrum of **3c.**

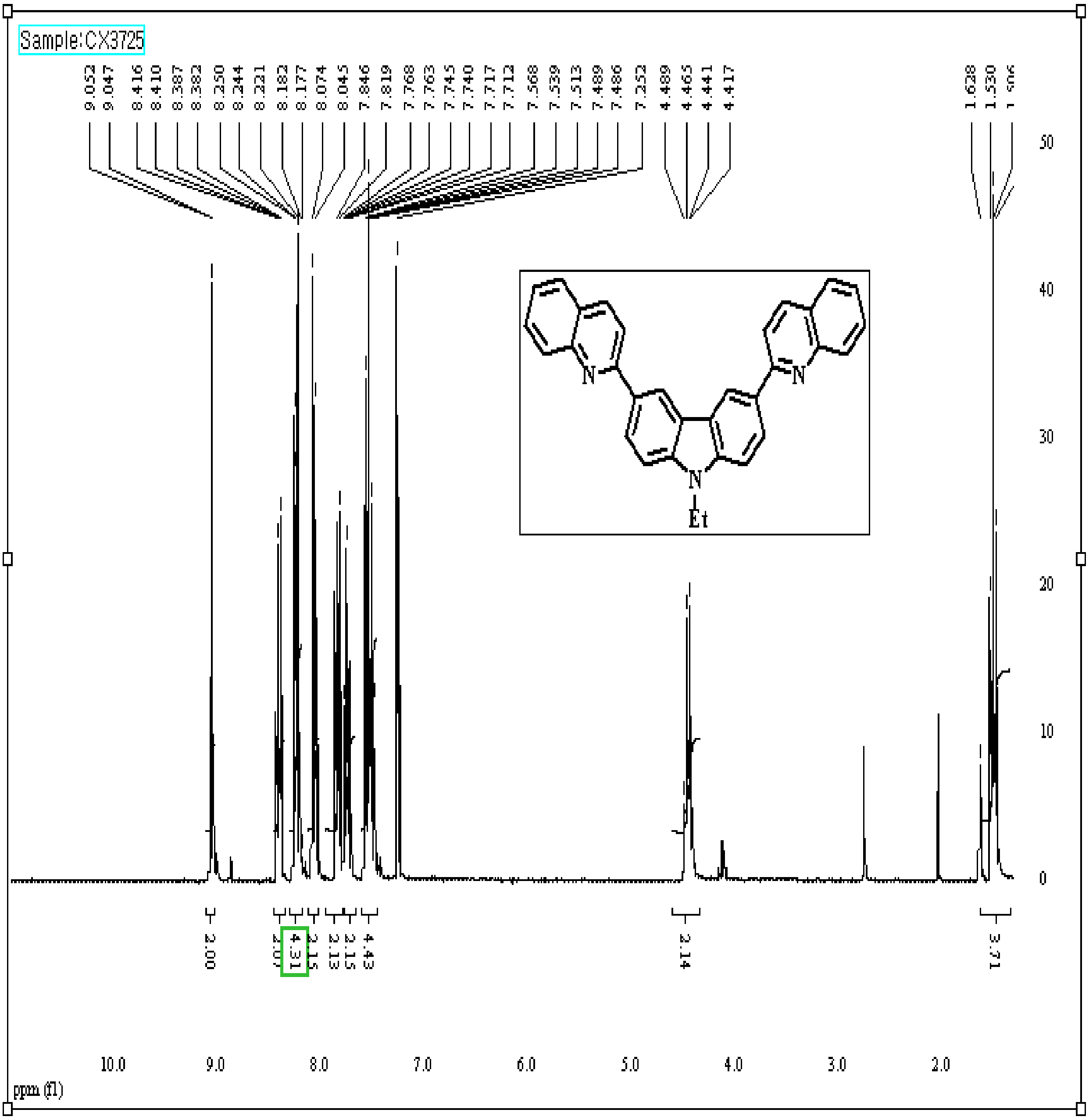

Figure S4: ^{1}H NMR spectrum of **5a.**

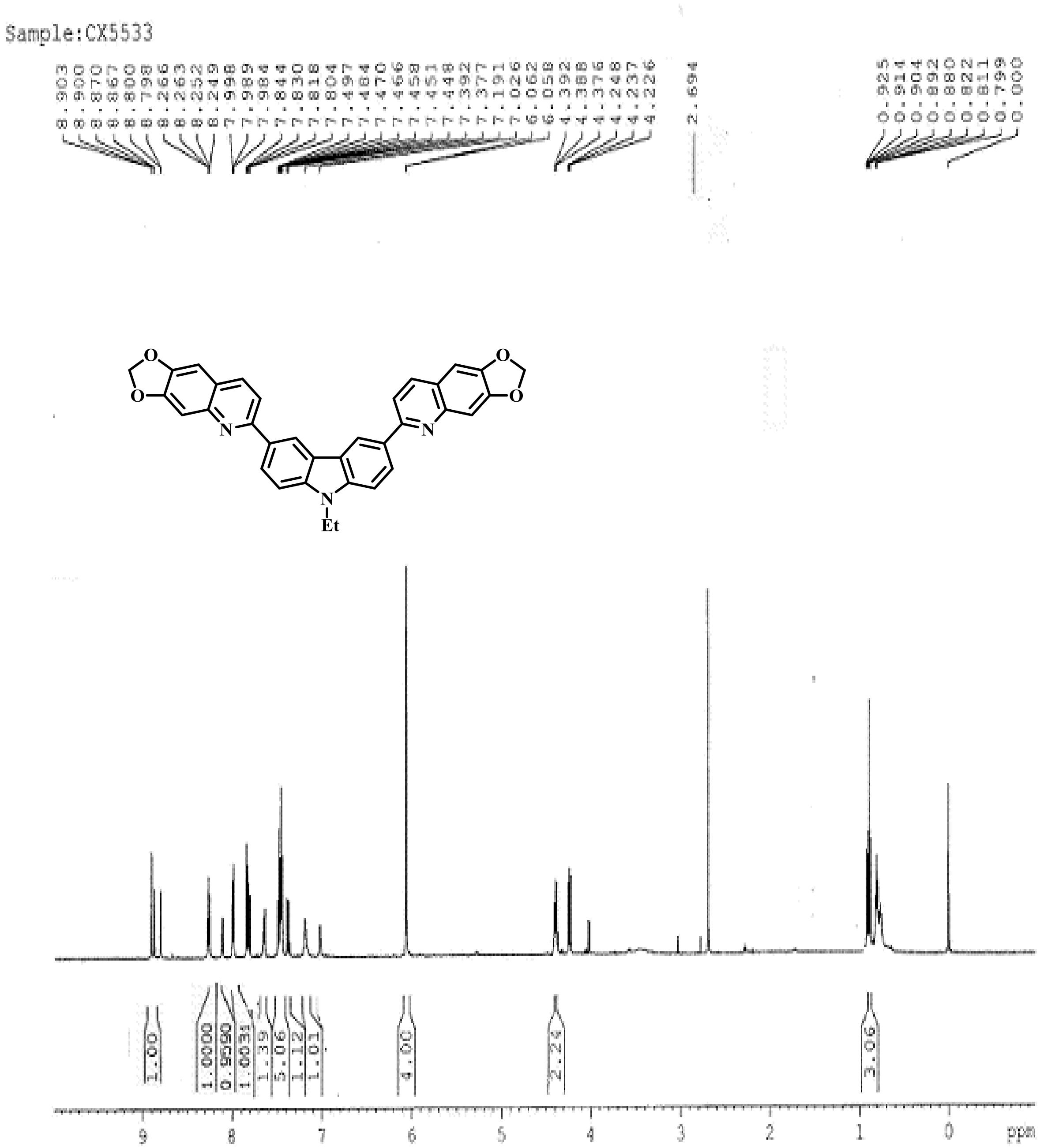

Figure S5: ^{1}H NMR spectrum of **5b.**

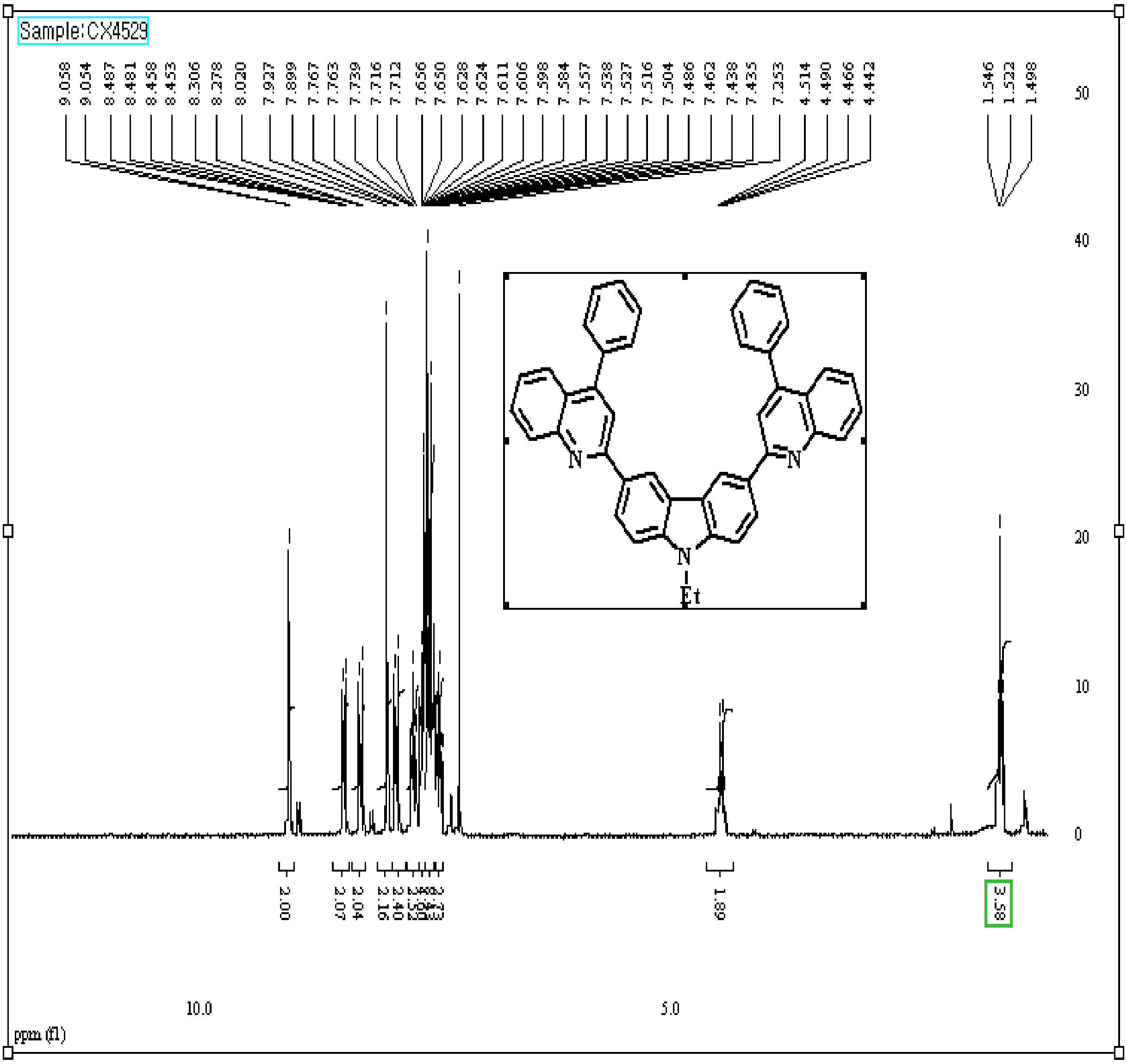

Figure S6: ^{1}H NMR spectrum of **5c.**
